# Towards a shared vision for research on evidence-informed policy-making

**DOI:** 10.2471/BLT.25.294211

**Published:** 2026-01-14

**Authors:** Bastien Kolt, Tanja Kuchenmüller, Ahmed Mandil, Annette Boaz, Daniel F Patiño-Lugo, John C Reeder, Kathryn Oliver, Robert F Terry, Sarah C Charnaud, Tarang Sharma, Tikki Elka Pangestu

**Affiliations:** a Science for Health Department, World Health Organization, Avenue Appia 20, 1211 Geneva 27, Switzerland.; b High Institute of Public Health, University of Alexandria, Alexandria, Egypt.; c The Policy Institute, King’s College London, London, England.; d Faculty of Medicine, University of Antioquia, Medellin, Colombia.; e London School of Hygiene & Tropical Medicine, London, England.; f TDR, the Special Programme for Research and Training in Tropical Diseases, World Health Organization, Geneva, Switzerland.; g Yong Loo Lin School of Medicine, National University of Singapore, Singapore.

The World Health Organization (WHO) defines knowledge translation as the exchange, synthesis and effective communication of reliable and relevant research results.^1^ Also known as research utilization or knowledge mobilization, knowledge translation is a transdisciplinary process that promotes sustained interaction between researchers and users, tailoring information so that evidence-informed policy-making interventions are more widely adopted.^1^

Despite considerable progress in knowledge translation and evidence-informed policy-making, translating evidence into policy and practice remains challenging, mainly due to insufficient institutional capacities and financial and human resources.^2^ Yet with the emergence of competing narratives and misinformation, the need for evidence-informed policy has become increasingly critical.

The concepts of knowledge translation and evidence-informed policy-making emerged in the 1980s from the evidence-based medicine movement, which sought to close the persistent gap between research and policy.^2^ WHO has played a central role in advancing these approaches. In 2005, WHO reformed its guideline production process, and the World Health Assembly adopted a resolution calling on Member States to strengthen the knowledge base for decision-making and establish mechanisms to support evidence-based policies.^3^ In response, WHO launched the Evidence-informed Policy Network, which has become a flagship initiative for advancing evidence-informed policy-making globally. The coronavirus disease 2019 (COVID-19) pandemic further reinforced this commitment, consolidated with the network’s 2021 call for action to enhance evidence-informed policy-making.^4^ Today, the network supports the institutionalization of evidence use in health policy-making in more than 50 countries.

A growing body of research on knowledge translation examines the methods, mechanisms and measurements that shape how evidence is produced, disseminated, used and can be promoted in policy-making.^5^ Much is already known about what works, including establishing relationships between researchers and policy-makers; timely access to high-quality and relevant research evidence; and capacity-building in evidence-informed policy-making for policy-makers.^6^ Using these lessons, along with systematic approaches and trusted partnerships, the network continues to promote the use of evidence for policy impact. For example, in Brazil, the network implemented a knowledge translation intervention, producing a policy brief and policy dialogues. The intervention led to new policy strategies that contributed to a rapid decline in perinatal mortality, demonstrating the tangible impact of evidence use and the network’s methods on health outcomes.^7^

Yet, despite widespread implementation, evidence on the effectiveness of knowledge translation interventions for policy-makers and on how they influence decisions remains limited. Persistent gaps remain in areas such as institutionalization of evidence-informed policy-making, raising awareness and co-creating policy-relevant evidence among producers, intermediaries and users.^8^ Knowledge translation research faces numerous challenges, with little consensus on the most effective methods for translating knowledge into policy across different contexts and settings. Knowledge translation research is fragmented and insufficiently coordinated within and across disciplines, sectors and countries.^8^ Expertise and funding in this field are often narrowly focused on specific priorities linked to funding agencies’ interests, resulting in missed opportunities for collaboration and in research waste.^9^ With the vast amount of research in progress, identifying true research gaps and prioritizing knowledge translation domains is challenging. Many of the efforts in prioritizing research in knowledge translation either target particular contexts or localities, or concentrate on specific sectors or institutions, resulting in further fragmentation.^10^ Moreover, they often focus on the relationships between evidence producers and users, neglecting the role and needs of other interest-holders, such as knowledge brokers, funders or educators. Globally coordinated knowledge translation research priorities are also absent in the literature, and knowledge translation research is disproportionately concentrated in high-income countries.^8^

To address these challenges, we saw a strategic role for WHO in convening evidence producers, users, intermediaries and funders to collaboratively set research priorities and catalyse investments to reduce research waste. WHO identified the importance of a multidisciplinary research agenda that addresses key gaps in how evidence informs policy and practice. This effort must be global and equitable to break silos, enhance research coordination, bridge disciplinary divides and enrich the current body of knowledge in this area.

Research agenda-setting is one of WHO’s core functions, and the Organization has a long-standing history in supporting Member States to identify priorities in health information and research.^11^ Since 2023, WHO has been leading a collective agenda-setting initiative with over 130 global experts to overcome these challenges by identifying research priorities and establishing a global research agenda that spans across sectors and disciplines.^12^

The process followed multiple stages and adhered to WHO’s guidance for research priority-setting exercises.^13^ The scope of this initiative is distinct from previous efforts because it focuses on research methods and on adopting a multisectoral perspective.

In the first stage, WHO commissioned an evidence map on the art and science of using evidence,^8^ and a literature review of previous knowledge translation research prioritization exercises.^10^ These reviews, complemented by a WHO global online survey in February 2024 with 153 respondents, resulted in an initial list of 120 research areas. A conceptual framework was also developed to guide the process.

The second stage involved expert consultations and a modified Delphi exercise to refine the list and build consensus on top priorities. An open call by WHO in December 2023 invited 151 vetted experts in knowledge translation and evidence-informed policy-making to participate, ensuring gender, geographic and professional balance across all WHO regions and country income levels. Online consultations and weighted scoring across Delphi rounds (March 2024–February 2025) narrowed down the list to 19 final research areas. Additional consultations with funders and United Nations agencies provided valuable feedback from a broad range of interest-holders.

The third and final stage, which is ongoing, focuses on disseminating the global research agenda and promoting its uptake among end-users through the development of context-specific local agendas, and translating priorities into actionable research projects. Engagement with researchers and funders to ensure alignment with global priorities is key. To initiate implementation, the Special Programme for Research and Training in Tropical Diseases launched a call for research proposals in May 2025, receiving 430 submissions; five projects were selected and will begin work in 2026.

The final 19 research areas are broad to ensure relevance across sectors and contexts. They cover both topics and approaches, offering a flexible foundation for context-specific research questions. An initial set of such questions, aligned with each of the 19 areas, was identified during the agenda-setting process and is detailed in the WHO project report.

The outcome of this process is a clear, prioritized list of knowledge translation research areas, endorsed by the global knowledge translation and evidence-informed policy community, to guide researchers, academics, funders, governments, civil society and international organizations in their knowledge translation research activities, strategic planning and funding decisions. 

The global research agenda aims to enhance our understanding of effective knowledge translation, raise awareness about research priorities and reduce research waste. These objectives will be supported by shifting focus from over-researched topics to those aligned with actual policy needs and evidence gaps and by strengthening coordination efforts, such as the Global Coalition for Evidence, that foster collaboration between researchers and funders. The agenda also helps identify where evidence exists and where further learning is needed. Focusing on priorities enables more impactful investments, ideally aligned with national or regional health and research priorities. The agenda also promotes complementarity among research areas to avoid duplication of effort. Finally, it serves as an advocacy tool to highlight the importance of this area and encourage governments and funders to allocate more attention and resources to these critical topics.

The strong participation and feedback received from experts involved in the agenda-setting process reflects a marked demand for a coordinated global effort to prioritize knowledge translation research. WHO envisions the global research agenda as a practical guide for knowledge translation funders, researchers and policy-makers to focus their efforts for maximum impact. To support regional- and country-level adaptation of the agenda, WHO will provide methodological guidance, facilitate shared learning and use its convening power to strengthen interest-holder engagement and visibility.

The research prioritization coincided with the launch of the Global Coalition for Evidence at the Global Evidence Summit in September 2024. Efforts are underway to integrate the research agenda within the Coalition’s mandate, where a dedicated working group will focus on translating the global research agenda into tangible research and coordinating engagement with the knowledge translation community. The coalition provides a sustainable platform for collaboration and convening with all relevant interest-holders.

To implement the global research agenda, we call on all actors involved in generating, prioritizing, funding and translating knowledge translation research to take several actions. First, join the Global Coalition for Evidence to advance this initiative and benefit from: (i) strengthened coordination around a shared agenda to ensure complementarity and avoid duplication; (ii) opportunities for global dialogue and peer exchange; and (iii) enhanced collaboration on joint initiatives and evidence-use projects. Second, align research strategies, activities and investments with the priority areas identified in the global agenda. Third, develop and support regional, national and subnational research agendas on knowledge translation and evidence-informed policy, using the global agenda as a reference. Finally, engage in cross-sectoral collaboration on knowledge translation research to create synergies and minimize research waste. Collective action on these priorities will help align investments, reduce duplication and enhance the use of evidence in policy-making.
